# Negative Life Changes in the Lives of a Working-Age Widows and Widowers and their Families After the Death of a Spouse- a Qualitative Study

**DOI:** 10.1177/00302228251328198

**Published:** 2025-03-20

**Authors:** Minna Hökkä, Anna Liisa Aho

**Affiliations:** 1 3831 Department of strategy, effectiveness and RDI, Diaconia University of Applied Sciences, Helsinki, Finland; 2 Faculty of Social Sciences, Nursing Sciences, University instructor, 7840 University of Tampere, Tampere, Finland

**Keywords:** widow, widower, family, life change, death

## Abstract

The aim of this study was to describe the negative changes in the lives of working-age widow (er)s and their families caused by the death of their spouse. A descriptive qualitative approach was used. The research data was collected with an electronic questionnaire. Negative life changes after the death of a spouse included increased relationship challenges, a cooling of family relationships, and a change in self-identity. In addition, loss of support and having to provide for basic needs alone, increased responsibility for children and everyday life. The feeling of life becoming burdensome were experienced. The death of a spouse causes negative changes in different aspects of life for working-age widow (er)s and their family. The results of this study can be used to develop societal sectors services and underline the particular importance of early support and of the negative life changes that widowhood can bring.

## Background

Close relationships are important to peoples mental and physical health (Sbarra, 2015). Married people have reported greater feelings of support, less stress and better well-being than single people (Diener et al., 2000; Walen & Lachman, 2000). In addition, research on young adults (18–25 years) shows that relationships provide companionship, promote health and reduce risk-taking behaviour (Beckmeyer & Cromwell, 2019; Furman & Collibee, 2014). Marriage provides security against unexpected experiences in sharing economic resources (Waite, 1995) and responsibilities and helping each other in difficult situations (Dehle et al., 2001). Therefore, losing a significant person through death is one of the most difficult experiences for a person to encounter (Shear, 2012). Grief is described as an individual and dynamic process that affects the bereaved and their life comprehensively and permanently (Hogan et al., 1996; Stroebe et al., 2001, 2007). In the lives of the bereaved, feelings and reactions related to grief manifest themselves sometimes more strongly and sometimes less strongly (Jacob, 1993). However, a deceased loved one can live on in the memories of the bereaved in a meaningful way through shared past, experiences, and memories for the rest of their lives (Dyregrov & Dyregrov, 2008). In addition to grief and longing for their spouse, widowhood involves a lot of adjustments and adaptation (Dunn, 2015).

After widowhood, life can feel unrealistic and restless, and things may not be under one’s control and the widows (er)s may become highly sensitive to perceiving everything around them (Blanner et al., 2021). Some of them may experience the death of a spouse as a relief, especially after a long-term illness (Blanner et al., 2021) or a bad relationship (Picazzio Perez Batista et al., 2019). The time and circumstances before the death of a spouse play a major role in shaping the widowed person’s life after the death of their spouse (Blanner et al., 2021). In addition, the quality of the relationship plays a role in widows’ survival, (Picazzio Perez Batista et al., 2019) and the widowed person’s needs to try to adapt to a new way of life after the death of their spouse. Thus, they have to start planning their life and future on a new basis. Widows may experience a sense of hopelessness about the future as they realise the limitations of life and become aware of their own impending death. (Chan & Chan, 2011).

For young widow (er)s, widowhood is often an unexpected event for which they are not necessarily socially, financially or emotionally prepared (Anderson et al., 2023; Haase, 2008). Working age in Finland is defined as the age between 15 and 65 years. In 2023, a total of 7325 working age persons died, of whom 4928 were men and 2397 women. In this study, working-age refer to widow (er)s aged 18–65. In Finland, the share of women in the labour force has grown ahead of men (Alho et al., 2024). After loss of a spouse, the widow (er) has the possibility to get financial support from the society (KELA, 2024) but there is no legal bereavement leave (Leysen & Wens, 2020). Widow (er)s of working-age are relatively young. Being widowed at young age affects many aspects of one’s life, when the loss of the spouse affects their finances, family roles, and working life (Anderson et al., 2023). In career theory in the transition model, becoming a widow (er), can be seen as a transition that changes the person’s role and responsibilities, that takes time for the transition to be successful, and that coping with the transition is different for each person. (Guichard & Lenz, 2005).

Widowhood is shown to be associated with mental health problems such as depression, sleep problems and anxiety, (Blanner et al., 2021; Kristiansen et al., 2019a, 2019b). Less attention has been paid to the impact on family members facing end-of-life challenges and death (Wang et al., 2024). The death of a spouse affects the whole family. Children are highly dependent on their parents for bereavement support and information about death (Menendez et al., 2020; Scott et al., 2019). At the same time, the bereaved face the challenges of being a parent to grieving children and continuing to live as a single parent (Glazer et al., 2010). Good social support contributes to well-being, especially for those who had a strong social support network prior to widowhood (Ungar & Florian, 2004).

In previous studies, the trajectory of medication use after widowhood has been explored (Maura et al., 2024). In addition, the focus has been on the prevalence of loneliness (Camacho et al., 2024; Kaishan et al., 2024), depression, anxiety, sleep problems (Blanner et al., 2021) or pain (Camacho et al., 2024) and experiences of widowhood (Chan & Chan, 2011). Research on young widow (er)s has focused on the association between the loss of a spouse with adverse health effects, such as an increased risk of chronic and acute diseases (Stroebe et al., 2017), cardiovascular diseases (Einiö & Martikainen, 2019), health changes (Kristiansen et al., 2019a, 2019b) and challenges (Taylor & Robinson, 2016). In addition, research have been made focusing on widow’s experiences (Ergün-Basak, 2024; Houston et al., 2016; Jones et al., 2019; Lamba, 2023; Lowe & McClement, 2011). Previous research has focused often on different age groups, for example, widowhood in older age, mostly over the age of 65. Research related to working-age widow (er)s are scarce, research on this age-group focus more on coping of different conditions such-as stroke (Šaňáková et al., 2024).

Qualitative studies of young widows’ experiences have been done in different countries as USA (Jones et al., 2019), Canada (Lowe & McClement, 2011), India (Lamba, 2023) and Nepal (Houston et al., 2016) Turkey (Ergün-Basak, 2024). Previous research has shown that widowhood affects the health and daily life of the widowed person, but knowledge of the negative life changes is still lacking. There is a gap in qualitative research on the negative impact of widowhood on the lives of working-age widowed persons and their families, especially from a Nordic perspective. It is therefore important, to explore this topic in order to gain knowledge that can be used to develop support and services for working-age widowed persons and their families. The purpose of the study was to describe the negative life changes caused by widowhood for working-age widows and widowers and their families, by using a descriptive qualitative approach. The following research question was sought to be answered in line with this purpose; what negative changes has the death of a spouse brought to the lives of working-age widow (er)s and their families?

## Methods

The method used in this study was a descriptive qualitative approach with an inductive content analysis (Kyngäs et al., 2019). The intention of the study was to present a comprehensive summary of the phenomenon of interest without claiming any methodological roots (Polit & Beck, 2020). The data were collected using an e-form from widow (er)s of working age who had lost their spouses. This study is part of a larger study related to widowhood.

### Participants

Participants were recruited by using a purposive sampling, which was chosen to find participants who are representative of the phenomenon of interest (Polit & Beck, 2020). The sampling was carried out through the webpage of the non-profit peer support association Nuoret Lesket Ry, and an email was sent to all the members of the association by the second author (ALA). The criterion for participation in the study was being widowed at working-age (18–65 years). A total of 96 eligible working-age widow (er)s participated in the survey.

The average age of the widow (er)s was 48 years. The average time since the death of the spouse at the time of response was 4.5 years. The average age of the spouse at the time of death was 48. The average number of minor children in the family (including children of the deceased spouse) was 1.5 (Table 1). The majority (89%) of the widow (er)s were women. More than half (65%) of widow (er)s worked full-time before the death of their spouse. Slightly fewer widow (er)s worked fulltime (55%) after the spouse’s death at the time of the survey. At the time of participating in this study, the marital status of the majority of the respondents (80%) was widow (er) (Table 2). Less than half (45%) of widows and widowers reported that the death of their spouse had occurred without prior warning, and a third (31%) of respondents had more than six months’ prior knowledge of their spouse’s impending death.

**Table 1.** Background Information About the widow (er)s and Their Family.

Demographic data	Mean	Range
Age of the widow (er)	48 Years	26–69 Years
Age of the deceased spouse	47.5 Years	25–69 Years
Number of underage children in the family	1.5 children	0-6 children
Time since becoming a widow (er)	4, 5 Years	3 months-21 years

**Table 2.** Background Information of the widow (er)s.

Background data	N (96)	%
Gender		
Male	10	10
Female	86	89
Age		
<30 years	3	3
30–40 years	17	18
>40–50 years	37	39
>50–60 years	31	32
>60 years	8	8
Education		
No vocational education	2	2
Vocational or other courses	1	1
Vocational degree	40	41
University of applied sciences degree	13	14
Academic degree	40	42
Working before widowhood		
Full-time work	62	65
Part-time work	6	6
Unemployed/laid off	4	4
Retired	3	3
Stay at home mother/Father	11	12
Student	5	5
Other	5	5
Working at the moment		
Full-time work	53	55
Part-time work	13	14
Unemployed/Laid off	9	9
Retired	3	3
Stay at home mother/Father	2	2
Student	4	4
Other	7	7
Marital status before widowhood		
Married	84	88
In a relationship	12	13
Current marital status		
Married	6	6
In a relationship	6	6
Unmarried	6	6
Divorced/Living separated	1	1
Widow (er)	77	80

### Data Collection Tools

The data was collected by means of an electronic questionnaire, the online survey tool Lime Survey was used to collect the data. The questionnaire was preceded by a cover letter providing information about the study. The questionnaire was developed by the research team and included the demographic information and open-ended questions related to widows’ and widowers’ grief, coping, life changes and changes in working life. The demographic information was the respondent’s gender, age, basic education, employment status, health experience, and current marital status. Background information (see Table 1) about the deceased spouse was also asked. The open question analysed in this study was: What negative changes has the death of your spouse brought to you and your family’s life?

### Data Analysis

The demographic information was statistically analysed by using SPSS for Windows 25. The open-ended question data was analysed using qualitative content analysis (Elo & Kyngäs, 2008). No theoretical framework was used as a starting point (Elo & Kyngäs, 2008; Kyngäs et al., 2019). The manifest content was included in the analysis, and words, sentences or phrases which constructed a meaning were used as the unit of analysis. (Elo & Kyngäs, 2008.) The analysis was carried out in four stages. First, the data were transcribed verbatim from the questionnaires into a Microsoft Word template. The researchers read through the data carefully to familiarise themselves with it. Secondly, the researchers identified expressions that corresponded to the research question. The data corresponding to the research questions were reduced to codes. The total number of reduced codes of negative life changes was 393. Thirdly, after the reduction phase, the reduced codes were grouped together based on similarities in content. In the fourth phase, the reduced codes grouped by similarities were abstracted. Reductions grouped in the same category were named with descriptive terms to subcategories. Similar subcategories were then further combined into categories and named with concepts describing the content.

### Trustworthiness and the Role of the Researcher

Trustworthiness in this study was strengthened when the method chosen was suitable for the purpose of this study. Credibility was enhanced by detailed reporting of the sampling, data collection and analysis process. Furthermore, dependability was strengthened by presenting the table of categories identified through content analysis (Table 3). Confirmability was strengthened by focusing on the manifest content so that the results would represent the views of the widow (er)s. The authenticity of the results was strengthened by providing citations from the authentic data. (Elo et al., 2014; Kyngäs et al., 2019).

**Table 3.** Negative Changes in the Lives of the Working-Age widow (er)s and Their Families Caused by the Death of Their Spouse.

Category	Subcategory
Increasing challenges in relationships	Challenges in a new relationship
	Lack of understanding from other people
	Decrease in the social life
	Reduction in circle of friends
	Feeling abandoned by friends
Challenges in family relationships	Increasing family disagreements
	Challenges with relatives
	Breaking off relations with relatives
Negative changes in identity	Difficulties to get used to the new widow (er) identity
	Loss of self-esteem
	Negative change in behaviour
Losing support and taking care for everyday life alone	Lack of the other to share common issues
	Loss of emotional support and physical contact
	Experiencing constant longing for the spouse
	Taking responsibility of the home alone
	Financial worries
Increased responsibility for the children	The challenges of children’s school related issues
	Children with complex symptoms
	Shifting too much responsibility to the children
	Difficulty of setting boundaries when raising the children
	Feelings of inadequacy when raising their children
	Lack of a second parent for the children
	Increased concern for children’s coping
Challenges in coping after the spouse’s death	A feeling of inadequacy of resources
	Suffering from the stressful life situation
	Reduction of personal time
	Increased mental fatigue
	Increased fears of death and loss
	Difficulty in dealing with grief
	Experiencing feelings of loneliness
	Feeling of disappointment with society
	Difficulty in planning for the future

The researchers discussed the analysis and findings throughout the analysis. The second author did the first reduction and coding of the data, the first author (MH) went through the entire process, and changes were made after discussions until a consensus was reached. No prior relationship was established between the researchers and the participants in the survey. The authors are experienced researchers and familiar with the phenomenon of interest. In terms of reflexivity, the authors are aware that this inevitably shaped all components of the research.

## Results

### Negative Changes in the Lives of the Widow (er)s and Their Families Caused by the Death of Their Spouse

Six categories emerged from the data, namely: increasing challenges in relationships, challenges in family relationships, negative changes in identity, losing support and taking care of everyday life alone, increased responsibility for the children and challenges in coping after the spouse’s death. Additionally, there are related subcategories that support these categories. All categories and subcategories are presented in Table 3.

### Increasing Challenges in Relationships

The participants experienced ***challenges in new relationships***. Some of them described a lack of interest in finding a new partner, and some widows described difficulties to start a relationship with a new partner. Some widows described feelings of disbelief in the possibility of falling in love again. They described challenges as getting into a bad relationship or experiencing various problems such as being mistreated and abused because of their own weakness. In addition, the widow (er)s described that they were afraid of getting involved in a new relationship and the possibility of losing a relationship again.

“I have not been able to move on with my life in terms of, for example, being interested in finding a new partner. I just miss my spouse, and I can't imagine anyone else replacing my spouse.” (Widowed at the age of 43, response 84)

“Fear in new relationships [exists] about whether that other person will also die soon - whether I dare to throw myself into this, in case I hurt myself again...” (Widowed at the age 28, response 34)

The widow (er)s described a ***lack of understanding from other people***, when other people do not understand their situation. Some widows felt that people belittled them or thought they were looking for pity from other people.

“I've also noticed that in the car trade, for example, a lonely woman is not taken very seriously, or at least my huspand used to get things done better.” (Widowed at the age of 39 years, response 12)

The widow (er)s described a ***decrease in their social life***. They felt themselves as different, isolated from social situations, and feeling left out among people. They experienced exhaustion in social situations, as well as a reduction in interaction and social relationships as a result of widowhood.

‘I feel alone, lonely, even though I sometimes see my friends... I would like to go to festivals and events with someone to share experiences.’ (Widowed at the age of 49 years, response 96)

The widow (er)s experienced *a* ***reduction in their circle of friends*** when as a result of widowhood their circle of friends was shrinking, and fewer meetings with family friends took place. Some of the widows described that only their real friends stayed with them.

“Friend couples have disappeared. (Widowed at the age of 43 years, response 51)

Widow (er)s described that they ***felt abandoned by their friends,*** which was accompanied by a feeling of being an outsider among their friends. Some widows perceived receiving empty promises from their friends as a negative experience, as also the loss of communication and meetings with friends, and the loss of support.

“Social relationships have almost completely disappeared, I have heard a lot of empty promises and wishes etc. Even with the remaining friends …. we don't communicate…, and with friends it can be months in between.” (Widowed at the age of 38, response 95)

### Challenges in Family Relationships

The widow (er)s described ***increasing family disagreements*** related to their widowhood. The family atmosphere became irritable, with arguments over trivial matters and an increase in fights within the family. In addition, some widows experienced a decrease in contact with their children, as well as an increase in tensions with the children, for example, arguing about their computer use.

“..the children don't communicate with each other, the youngest doesn't talk about the lost parent... I'm still trying to replace what my spouse does and that's why I can't do it sometimes because teenagers are too lazy to do anything before I get angry or cry”. (Widowed at the age of 44 years, response 83)

**C*hallenges with relatives*** after the death of their spouse were expressed by the widow (er)s. This experience was accompanied by a loss of being around with relatives and a decrease in communication with them. In particular, with relatives of the deceased spouse, they became more distant or communication with them became one-sided and meetings decreased. Some of them described disparaging behaviour and disappointment in the behaviour of relatives. Some widows expressed that there was a lack of shared grief among relatives, and that the deceased spouse was seen as perfect. In addition, some widow (er)s expressed that the relations and communication with in-laws became more difficult. This appeared as a blaming of close relatives, neglect of one’s own mother, or even wishing for the death of one’s own sick mother. In addition, widow (er)s experienced a lack of interest from the deceased spouse’s family, dwindling relations with relatives, and a bitterness towards relatives.

“There has been very little contact with relatives on the spouses's side. They themselves are not active in keeping in touch, and contact is entirely my responsibility.” (Widowed at the age of 32 years, response 26)

“I can no longer care about my own parent who lives in a nursing home. (The parent) has late-onset Alzheimer's.” (Widowed at the age of 44 years, response 88)

Some of the participants described that the death of their spouse led to a ***breaking off of relations with relatives***, which appeared for example as a complete exclusion of the children of the deceased spouse from the widows’ lives. The widow (er)s relations with relatives of the deceased spouse could also break off as a result of disputes or excessive advice from relatives, for example about childcare.

“Some of my spouse’s relatives have been cut off and I am somewhat sad about this, although I have got used to it.” (Widowed at the age of 34 years, response 63)

### Negative Changes in Identity

The participants described **difficulties to get used to the new widow(er) identity.** They described a loss of their previous identity, their current identity was only linked to the death of the spouse, and that they had to recreate their identity completely. The widow (er)s found their change in identity painful and had to rethink their self-image. Moreover, they had to get used to their new marital status and their role as widow (er)s.

“The loss of my own identity is a painful change in that I am odd, while other acquaintances and friends remain even.” (Widowed at the age of 43, response 85)

The ***loss of self-esteem*** experienced by the participants included feeling low self-confidence and suffering from low self-esteem. Some of the widow (er)s experienced inferiority as being the odd one out, which was associated with their experience of inequality compared to other families and of being left alone. Some widows expressed also feeling themselves less of a mother than before widowhood.

“My identity has taken a big hit, I feel like a freak, my self-confidence is low**.”** (Widowed at the age of 42 years, response 55)

A ***negative change in behaviour*** was linked to the experience of a hardening of their character. Some of the widow (er)s described changes as becoming ruder, less complacent, more impatient, cynical, short-tempered, unable to concentrate, or being quiet in company. Some of the widows felt more tearful or suspicious towards other people than before. Drinking alcohol or not taking up hobbies were also described as changes in behaviour after the death of their spouse by some widows.

“I've become a bit blunt, maybe, because I just don't have the energy to make small talk or meet many new people. In some social gatherings (parents' evening, etc.) I'm quite quiet and an outsider”. (Widowed at the age of 37 years, response 23)

### Losing Support and Taking Care of Everyday Life Alone

The participants experienced a ***lack of another to share common issues with***. This was related to missing things they had done together with their spouse. They negatively experienced the absence of a partner to talk to, with whom they could share their thoughts, hopes, fears and dreams. The loss of everything they did together following the death of their spouse was especially negative.

For my part, .... the absence of another person to share things with .... We were always Us, did everything together, dog walking, bike rides, gardening. A lot changed after the spouse left. (Widowed at the age of 57 years, response 52)

“My spouse was the only person in my life with whom I shared my thoughts, hopes, fears, dreams. I am mentally and emotionally lonely”. (Widowed at the age of 44 years, response 10)

The participants experienced a ***loss of emotional support and physical contact***. The participants described that they lacked a friend and a spouse in their lives. Some widows described how, with the loss of their spouse, there had also been a loss of energy, joy and positivity in their lives. Some widows expressed feelings of a lack in physical intimacy.

“There is no spouse. … The joy and positivity that a spouse brings has been lost. So has the energy the spouse brought to everyday life.” (Widowed at the age of 35 years, response 80)

The participants ***experienced constant longing for their spouse***, and the silence in the home and especially during the holidays, was seen as a negative aspect. In opposite some of the widows were longing for own time and space during the holidays. Some of the widows described feelings that the marriage was unfinished and that they therefore missed their spouse immensely. The widow (er)s also expressed feelings of abandonment.

“Holidays are difficult - I would like to be all alone, [but] the family does not want to be left alone.” (Widowed at the age of 50 years, response 34)

The participants described an increasing **responsibility for the home alone**, with increasing household chores and the learning of new skills seen as negative changes. They expressed feelings of getting tired of keeping the home tidy. When they lost their spouse, some widows expressed that they had to carrying on unfinished projects alone, taking on the tasks and role of the deceased spouse, and taking care of things that they felt did not belong to them.

“More housework and having to learn new skills....” (Widowed at the age of 45 years, response 60)

“I still try to replace my spouse’s tasks …” (Widowed at the age of 44 years, response 64)

The widows also described ***financial worries*** which affected their ability to manage everyday tasks and to cope. Widows used their energy to reflect on financial worries, as well as describing financial changes and struggles.

“I can't keep my house tidy when I have to think about money all the time” (Widowed at the age of 45 years, response 9)

### Increased Responsibility for the Children

The participants perceived ***the challenges of children’s school related issues*** as negative changes. These issues included children’s lack of interest in school, difficulties related to school mornings, and concentration problems at school. For adolescent, for example, this could be manifested in inability to continue their studies. Some of the widows felt that the traumatisation of their children made it difficult for the children to go to school.

‘(Child) was very traumatised by the death of his father and has had difficulty coping at school.’ (Widowed at the age of 38 years, response 36)

“The (child) moved back home and is unable to concentrate on studies.” (Widowed at the age of 55 years, response 50)

Some of the widows described that their ***children had complex symptoms*** which included abdominal pains, attachment to adults, aggression, stress, and a lack of communication. Children’s symptoms also included feelings of guilt, traumatisation, self-esteem problems, rage, and self-blame for the death of a parent. Children could become depressed and lack a sense of joy in their daily lives. According to the widows, the virtual world could become a substitute for reality for their children. They were also more nervous about new places and people than before, and widows felt that it was difficult for the children to deal with their grief, and that they lived in constant fear of losing other people or the other parent.

“We talked a lot, we went through death and the emotions associated with it, such as sadness, fear, anger and guilt. The sadness and anger are expressed as aggression in the older child, and the guilt is expressed as self-esteem problems.” (Widowed at the age of 41 years, response 11)

Widows shared their experience of ***shifting too much responsibility onto their children*** as a negative change, where the children had to protect and take care of the surviving parent, this was expressed in families with underaged children and in families where the children were already adults. Widows expressed that the underaged children and adolescents became too helpful and expressed feelings of emotional burden. Widows also experienced an excessive dependency on their children, and a relationship that became too close. Linked to this, widows also felt that children had to grow up and become independent too quickly.

“I worry about the children when they try to help too much, and thus sacrifice their own lives for the sake of their parent” (Widowed at the age of 61 years, response 56)

I am the only parent to the children, and it weighs on my shoulders… Fortunately, as the children have grown up, they have had to take on some of the tasks but even that is not quite right. (Widowed at the age of 40 years, response 12)

Widow (er)s felt that it had become more ***difficult to set boundaries when raising their children***. Some widows expressed that they tended to bend the rules and spoil their children more in the absence of the other parent. Widow (er)s found it challenging to act decisively without another adult, and resolving conflicts was challenging. They felt that being a single parent meant that they did not have the same time or energy to manage their children’s affairs as before. Widows expressed that they were sometimes also overlooking their children’s issues, and experienced fatigue in setting limits for their children.

“As a single parent, you don't always have the time or energy to intervene in what your children are doing/not doing, and you have to turn a blind eye to some things. You don't always have the energy to start wrestling with teenagers.” (Widowed at the age of 46 years, response 74)

The participants described ***feelings of inadequacy when raising their children.*** They felt tired of being the only adult to raise them and often felt that everyday life had become a struggle to cope, lacking outside help. They found it particularly challenging to bring up a child in adolescence without a second parent. Some widows said that everyday life became too busy, binding and burdensome as a single parent. It was difficult to find time to support the child’s well-being, and to engage in hobbies and support the child’s schooling. In particular, widows experienced inadequacy in meeting the needs of their adolescent children and also fatigue with the laziness of teenagers. They furthermore experienced a lack of strength to support their children, and a lack of energy in even being with their children.

“When my (child) was going through puberty, I really wished that the missing parent had been involved in all the day-to-day activities and decisions. But in the end, others don't understand things in the same way.”(Widowed at the age of 53 years, response 14)

The ***lack of a second parent for the children*** led to situations where the children sometimes lacked a role model in social situations. In particular, children lacked the attention, presence and support of the other parent. The children’s family life was also perceived to have been reduced in the absence of a second parent, and some female widows felt that their sons lacked a male role model and the boys experienced fatherlessness in their lives. The widow (er)s also had to make decisions alone and felt that they needed another adult to share responsibilities. Overall, children experienced difficulties and unpleasant situations in their lives because of the loss of their other parent and felt different and inferior because of this. The widows whose children were in young adulthood expressed concern about their children’s ability to build their own family lives as the grandchildren lacked a second grandparent.

“I experience inadequacy in relation to my children who are adolescents. They lack the other parent's gaze, attention and all the presence they used to get. I can't make up for it by having to deal with so many more practical things than before.” (Widowed at the age of 43 years, response 85)

The widow (er)s felt that an ***increased concern for children’s coping*** fell on them, and they were concerned about their children’s coping in life whether their children were minors or adults. The widows expressed concerns about the moods and skills that underaged children and adolescents develop following a traumatic experience.

“I am concerned about the children's survival in the future. I am worried about how much they have suffered and are suffering from the death of their parent, and how much I have contributed to their grief by not daring to help them express their feelings or other concerns themselves.” (Widowed at the age of 44 years, response 83)

### Challenges in Coping after the Spouse’s Death

The widow (er)s expressed ***feelings of an inadequacy of resources.*** They described a reduced ability to cope, feeling that their other capacities are inadequate, and also a reduction in their own strength. Widows also experienced a loss of resilience.

“ ...my energy resources are often very limited and social situations with people other than a couple of significant others are exhausting” (Widowed at the age of 37 years, response 23)

***Suffering from the stressful life situation*** was described as a tiredness in claiming their own rights. Experiences of having to fight for one’s own space was also perceived as a negative aspect by some widows. Some widows felt stressed and poor in every way, where everyday life felt constantly busy, with a lot to remember and think about. Some widow (er)s felt stressed about looking after the health of other family members at the stressful life situation.

“It is very hard to be the sole carer of three school-age and adolescent children. There is always a lot to do and a lot to remember, which causes stress and arguments.” (Widowed at the age of 41 years, response 55)

The participants experienced ***a reduction of personal time.*** As a result of becoming widowed, they found it difficult to meet their friends and pursue their own hobbies due to a lack of time**.** The widows also experienced ***increased mental fatigue***, which included the experience of being rejected and stigmatised. Overall, widows felt tired and this tiredness was directed at their children, with increased insomnia and unpleasant thoughts intruding at night.

Loneliness prevents you from even trying. I was suffering from insomnia before and it just keeps on happening, especially at night when all the thoughts come into my head. (Widowed at the age of 57 years, response 3)

***Increased fears*** of losing a loved one appeared as a salient aspect. Some widowers described that they were cutting back on personal appointments because of fear. In particular, they felt fear of their own death and leaving their children alone. Some widow (er)s expressed that widowhood concretised the fear of ‘terrible things’ and the possibility of them happening.

“I am afraid that something will happen to myself (and the children will have no one), and because of this I have even had to cut back on my own personal appointments.” (Widowed at the age of 44 years, response 36)

Their ***difficulty in dealing with grief*** included suppressing their own emotions. Some widows described difficulties in dealing with their own grief alongside their children’s grief, as well as describing emotional pain. The widow (er)s also described the ***experience of loneliness***, which was associated with feeling and experiencing increasing loneliness, feeling lonely in the company of couples, feeling lonely in everyday life. Some widows described they felt lonely when their children moved away.

“I've been very strong at times, because I've had to deal with my own grief as well as my children's...” (Widowed at the age of 41 years, response 11)

***Feelings of disappointment with society*** included widow (er)s’ experiences of mistrust and injustice towards health and social services, with a perception of social services as incomprehensible. In addition, some widows described a sense of bitterness towards society, and feelings that widowhood made it difficult to find a job and a place to study.

“Distrust of health care, it's a guessing game. Distrust of the social services and their total lack of justice and understanding, only added to the pain and distress of our family. Bitterness towards society, creating pressure on an already heavy burden bearer.” (Widowed at the age of 44 years, response 64)

Compounding the ***difficulty of planning for the future*** was the participants’ experiences of struggling with uncertainty. Some widows expressed a difficulty of continuing with life because of its uncertainty. Particularly, it was felt that it was difficult to overcome the tragedy, and planning for the future was perceived as an insurmountable difficulty.

Sometimes difficulty in pushing for goals in a world where anything can happen to anyone at any time. (Widowed at the age 46, response 4)

“In one second, all the plans for the future that we had made together were stripped away.” (Widowed at the age 61, response 56)

## Discussion

The death of a spouse was related to many negative life-changes for the working-age widow (er)s and their families. First of all, the widow (er)s expressed that their social network changed after the death of their spouse. Previous studies have reported similar findings (Dunn, 2015; Jones et al., 2019; Lowe & McClement, 2011). In addition, in this study they described challenges in relationships and felt abandoned from their friends. They expressed lack of support from friends and relatives. This finding is an important insight when, social support has been reported to protect from the negative effects of bereavement and facilitated positive adjustment in younger widow (er)s (Anderson et al., 2023). In the developed countries the life expectancy has increased and is forecasted to still improve in the future (GBD 2021 Forecasting Collaborators, 2024). When people are expected to live longer, and the context of ageing population is prevailing it may increase the feelings of unpreparedness and lack of social support in a situation where a person in working-age dies. This is particularly emphasized in sudden deaths of working-age people due to unpredictability, in which case widow (er)s have not been able to prepare for the loss, which may lead to, for example, prolonged grief disorder, post-traumatic stress disorder, or depression (Kristensen et al., 2012). It is important that professionals provide support for widow (er)s, especially as previous research has shown that although many widows were successful in developing their social support, psychological and emotional problems are common (Petrus et al., 2016).

The research results showed that the widow (er)s described a lack of confidence in the future and a fear of losing the new partner, which challenged the formation of a new relationship. This was an aspect that was less frequently addressed in previous studies. However, according to Chan and Chan (2011) widows may experience a sense of hopelessness about the future as they realise the limitations of life and become aware of their own impending death. It is very natural to contemplate and even fear one’s own death after the death of a loved one. Losing hope in life is something for which grieving widow (er)s may need professional help to avoid the negative consequences of grief, such as suicidal thoughts and actions (Stroebe et al., 2007).

Widow (er)s described negative changes in their identity and difficulties in building their new role, a finding consistent with previous research (Lowe & McClement, 2011). In the working age, the expected role was to work or look after the family and live as a couple. The loss of the spouse broke these expectations, and the widow (er) had to rebuild their roles and identity. This was seen as a negative life change and difficulties in building the new role and identity were described. Similar findings were reported in an earlier study describing the challenges of identity change for younger widow (er)s, when in society widowhood is more commonly associated with older age (Anderson et al., 2023). They also described negative behavioural changes, including increased alcohol consumption. A previous study also showed that widows increased their alcohol consumption more than the control group after the death of their spouse (Kowalski & Bondmass, 2008). These findings help to understand the challenges faced by the widowed and can help professionals to better target support to widow (er)s.

In this study losing support and taking care for everyday life alone was negative changes that the widow (er)s experienced. Surviving alone with every-day life, worries about money, and losing a partner with whom to share common moments were particularly difficult. Similar issues have also been described in earlier studies, (Bellamy et al., 2014; Picazzio Perez Batista et al., 2019) and the need for financial support has been raised as a difficult and worrying aspect (Picazzio Perez Batista et al., 2019), that should be considered by professionals, (DiGiacomo et al., 2015) especially when it has been shown that widows who had more financial worries also had more grief (Dunn, 2015).

When being a widow (er) in working-age the likelihood to also have children or ageing parents in need of assistance is bigger than in older age. The widow (er)s in this study reported challenges in family relationships, they described increased disagreements in the family and reduced or even breakdown in relationships with relatives. At the same time, they described the increased responsibility for the children or own parents in need for assistance. Previous studies show that widows who are left as sole caregivers are characterised by coping with their own grief at the same time as caring for grieving children (Werner-Lin & Biank, 2012; Yopp et al., 2015). They also described the difficulty of caring for other family members when they needed support for themselves, similar to findings of previous research (Ungar & Florian, 2004). Children who have lost a parent are at risk of poor psychological outcomes including depression, anxiety and somatic complaints (Brent et al., 2012). At the same time as widowhood may have increased the narrowing of the social safety net, it has also increased challenges to cope with responsibilities to care for children and family members. As also stated in earlier studies, the need to develop tailored support for widow (er)s in young age is important (Anderson et al., 2023). The support should take into account the individual needs of the widow (er) and the family members.

Challenges in coping after the spouse’s death was described by the widow (er)s. According to this research, widow (er)s perceived a quiet home as negative aspects. This is in line with earlier research which found that widows faced challenges, such as long time periods spent alone and a lack of things to do (Chan & Chan, 2011). In addition, in this study they described mental fatigue, loneliness and difficulties to cope with grief. Depression (Jones et al., 2019), significant negative impact on widow (er)s’ mental health (Blanner et al., 2021) and loneliness has been reported in earlier studies (Brown et al., 2018; Camacho et al., 2024; Lamba, 2023; Vedder et al., 2024). The widows in this study experienced also belittlement, as also Haase (2008) has mentioned that young widows felt they had no right to grieve as they had the opportunity to remarry and live further a long life. Also, the working-age widows described a lack of free time, which led to a reduction in opportunities to pursue their own hobbies. This should be taken into account when developing support, especially when widows who reduced their leisure activities following the death of their spouse have been reported to have more symptoms of depression than widows who continued to participate in leisure activities (Janke et al., 2008).

A significant finding in this study was the strong impact of widowhood on parenting and worrying about children and their various issues and symptoms. Consequently, it is important for health care staff to talk to widow (er)s about their support networks, and where appropriate, signpost them to services and resources where they and their children can get support. The many changes in their daily lives require multiple ways of supporting the whole family. So, health care and policy should take into account the negative life events associated with widowhood identified in this study, in order to provide widow (er)s and their families with the support they need.

### Strengths and Limitations of the Study

The participants represented working-age widow (er)s, and the data were rich, which strengthens the value of the results. To our knowledge, this is the first qualitative study to explore the negative experiences of widowhood in Finland. The findings are a valuable addition to the ongoing discussion about how best to meet the needs of widow (er)s and their families. In addition, data saturation was achieved during the analysis, which can be seen as a strength of the study (Saunders et al., 2018). The research also included certain limitations. For instance, the questionnaire was answered anonymously, therefore, it is impossible to know the reason(s) why certain widow (er)s refused to answer this question. There was also no opportunity to ask widow (er)s further questions or to return the results to them for comments or corrections. Another limitation to transferability is that most of the participants were women.

### Research Ethics

The subject of the study is sensitive due to the difficult life situation of the research participants. As there is little information available on the subject, the study topic was ethically justified. At the beginning of the electronic questionnaire, a cover letter explained the aim and process of the study, the voluntary nature of participation, and the confidentiality afforded to the participants and data (Bengtsson, 2016) which included that the participant could choose to withdraw from the study if they so wished. No personal data were collected in the survey, and any identifying information was anonymised. The contact details of the researcher were provided in the cover letter in case the participants wished to contact the researcher (TENK Finnish National Board on Research Integrity, 2023). Responding to the questionnaire was taken as informed consent. The study followed the principles of research ethics in regard to reliability, honesty, respect and accountability (All European Academies, 2023; TENK Finnish National Board on Research Integrity, 2023). Permission for the study was obtained from the Nuoret Lesket Ry association.

## Conclusions

In conclusion, the death of a spouse causes many negative life changes for widow (er)s and their families in many different areas of life. Widow (er)s felt that their lives had become burdensome, and the death of their spouse had left them alone with a heavy responsibility for their home and children and other family members. The children displayed various symptoms related to the loss of a parent. The results of this study confirmed that the death of a spouse has a negative impact on the widow (er)’s identity, relationships, and triggers emotional problems that among other things may also hinder their coping. As a topic for further research, it would be important to investigate how working-age widow (er)’s and their families are concretely helped after the death of their spouse.
